# Anamnesis Checklist for Informed Oral Contraceptive Choices: A Spanish Perspective

**DOI:** 10.1089/whr.2024.0073

**Published:** 2025-01-13

**Authors:** Josep Perelló-Capo, Ángeles Blanco Molina, Javier Trujillo Santos, María López Menéndez Arqueros, Esther de la Viuda, Borja Otero García-Ramos, Dolores Pérez Jaraíz, Luis Ignacio Devesa Otero

**Affiliations:** ^1^ Department of Obstetrics and Gynaecology, Hospital de la Santa Creu i Sant Pau, Barcelona, Spain.; ^2^ Department of Paediatrics, Obstetrics and Gynaecology, Preventive Medicine and Public Health, Universitat Autònoma de Barcelona, Barcelona, Spain.; ^3^ Department of Internal Medicine, Hospital Universitario Reina Sofía, Córdoba, Spain.; ^4^ Department of Internal Medicine, Hospital General Universitario Santa Lucía, Universidad Católica de Murcia, Murcia, Spain.; ^5^ Department of Gynaecology and Obstetrics, Complejo Asistencial Universitario de Palencia (CAUPA), Palencia, Spain.; ^6^ HM Gabinete Médico Velázquez, Madrid, Spain.; ^7^ Clínica Palacios, Madrid, Spain.; ^8^ Department of Obstetrics and Gynaecology, Hospital de Cruces, Universidad del País Vasco, Bilbao, Spain.; ^9^ Hospital Clinica Tara, Taraconte, Spain.; ^10^ Hospital Nuestra Señora de Fátima, Vithas Vigo, Vigo, Spain.

**Keywords:** oral contraceptives, contraception, progestin-only pills, anamnesis, safety, venous thromboembolism

## Abstract

**Objectives:**

This study aimed to develop an anamnesis checklist for oral contraceptive (OC) choice focused on their safety profile and associated risk factors.

**Study Design:**

This study involved eight health care professionals in Spain, including six gynecologists and two internists, selected for their expertise in contraception counseling. We employed the design-thinking process, structured in five phases: empathizing with patients’ needs, defining key areas of impact, devising innovative solutions, prototyping ideas into testable proposals, and validating prototypes. This process involved an analysis of the available literature, online discussions, and an online survey to evaluate importance and frequency of variables in anamnesis. Medians were computed for each variable, and the study group collaboratively determined the variables to include in the anamnesis checklist.

**Results:**

Women must be informed about contraceptive options, according to health care professionals. Body mass index, smoking status, blood pressure, and personal history were identified and prioritized as variables to consider during OC counseling. Participants emphasized the need to individualize the treatment, highlightling the safety profile of progestin-only pills over OCs due to the lack of increased venous thromboembolism risk.

**Conclusions:**

The study emphasizes the importance of an anamnesis prior to prescribing an oral hormonal contraceptive, as well as the most relevant risk factors that should be analyzed. A checklist was developed to facilitate safe OC prescribing.

## Introduction

The use of combined oral contraceptive (COC) pills containing a progestin and an estrogen has been widespread. In general, these medications are effective and considered safe, but their use in certain medical conditions may be contraindicated. The evidence suggests that the use of estrogen-containing contraceptives correlates with an increased risk of venous thromboembolism (VTE). This risk is influenced by numerous factors such as age, smoking, high body mass index (BMI), migraines, family history of VTE, genetic factors or puerperium,^1–5^ which should be considered before any recommendation. The incidence of VTE increases from 1–5/10000 woman-years in nonusers of COC to 3–15/10000 woman-years in users. However, this incidence is estimated to be 5–20/10000 woman-years in pregnancy.^6^

A safer alternative to COC are progestin-only pills (POP), which do not increase the risk of VTE.^7^ A new 4 mg drospirenone (DRSP) 24 + 4 pill has recently been introduced with a similar contraceptive profile and a good safety profile.^8,9^ Moreover, DRSP is not associated with an increased risk of VTE, myocardial infarction, stroke, hypertension, or hyperkalemia and does not affect the coagulation factors dependent on the liver,^9–13^ making it available to a wider population.

The Observatory of Sexual and Reproductive Health of the Spanish Society of Contraception reported that, in 2022, 75.7% of Spanish women of fertile age used any form of contraception and only 0.8% used POP, whereas 17% used COC.^14^ Classically, POP used to require a strict schedule and could cause unscheduled bleeding,^15^ but POP developed in recent years changed this situation. DRSP has a less restrictive timeframe, maintaining the inhibition of ovulation even after a 24-hour delay compared with the previous 12-hour window of desogestrel.^12^ Moreover, a reduction in unscheduled bleeding over time has been described for DRSP.^16^

Moreover, the main reason to avoid hormonal contraceptives in Spain (41.2%) was their safety and adverse events.^14^ Cardiovascular disease is a leading cause of death in users, and several factors are known to increase its risks, such as hypertension, dyslipidemia, diabetes, obesity, diet, smoking or an unhealthy lifestyle, childbearing age, or the use of COC. The prevalence of some of these factors is increasing with the current lifestyle, such as obesity, hypertension, or cigarette smoking.^17–21^

Taking into account all these factors and given the range of oral contraceptive (OC) options available, the choice of the recommended OC in each case should be based on their safety profile and the potential users’ needs. If the user’s risk profile is high, COC should be avoided, and POP should be the OC of choice. Despite this, there are no clear international guidelines because circumstances in each country differ; thus, all countries are expected to develop their own protocols for anamnesis, the collection of the medical history for evaluation.^22,23^ To facilitate this choice, the WHO Medical Eligibility Criteria for Contraceptive Use aimed to establish anamnesis recommendations for the consideration of various contraceptive methods focused on their safety profile.^24^

In this context, the role of the gynecologist is crucial in anamnesis, detecting potential risk factors for VTE and counseling potential users about the different OC options. This will allow the best OC to be chosen based on situation and safety profile. The aim of this study is to facilitate an adequate anamnesis checklist, facilitating the choice and counseling of those seeking advice about the safest OC based on their medical conditions and individual needs.

## Methods

### Participants

To create a checklist for contraceptive counseling, eight health care professionals from eight sites across Spain (Barcelona, Bilbao, Cordoba, Madrid, Murcia, Tenerife, Valladolid, and Vigo) participated in the study, providing a representative sample of the national territory. All health care professionals were chosen based on their experience with oral contraception counseling. The study group was composed of six gynecologists and two internists. The work sessions were held between December 2022 and January 2023.

### Design-thinking process

Design-thinking techniques were used.^25,26^ The design-thinking process is divided into five phases: (1) empathize—seek to understand what would be truly relevant for the users and gain insights into their needs; (2) define—consider the information gathered and use it to identify key areas where the biggest impact can be made; (3) devise—begin to create solutions to specific problems generating innovative ideas that can solve the users’ needs; (4) prototype—turn ideas into proposals, creating prototypes that can be tested in the real world; and (5) test—validate the newly-created prototype.

### Project phases

**FIG. 1.**
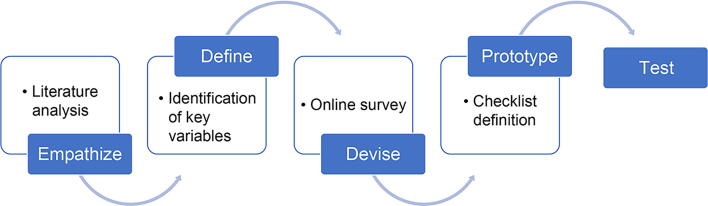
Design thinking process.

This project aims to determine an anamnesis checklist for choosing an OC following the design-thinking process (Fig. 1). Firstly, available information and literature were analyzed to completely understand the current situation. Afterward, the situation was further analyzed in an online work session, identifying key relevant aspects for the anamnesis that must be considered based on previous studies^17^ and the participants’ experience, creating a list of potential variables to be taken into account as part of an ideal anamnesis. After this first session, each member of the study group filled out an online survey focused on the previously identified variables to be included in an anamnesis, based on their perception of routine clinical practice. The survey included 21 variables to be scored based on a Likert scale on their importance (1—unimportant, 4—very important) and the frequency during anamnesis (1—rare, 4—very common) (Supplementary Table S1). Medians were calculated for each variable as a measure of central tendency and the obtained results were shared with the participants. During the phases of the project, variables were identified, prioritized, and discussed. The variables evaluated by the professionals are presented in Supplementary Table S1. The selected variables covered different areas, including family and personal history, previous conditions, and life habits. Given their different perspective, to ascertain whether the internists’ contributions were altering the median score obtained for each variable, we analyzed the scores given by gynecologists only (*n* = 6).

Afterward, the study group met in another online work session to discuss the results and select the variables that should be present in an anamnesis checklist, based on the survey results, their own criteria, and the known risks of VTE. These variables were grouped into five categories to facilitate the implementation of this checklist.

## Results

**FIG. 2.**
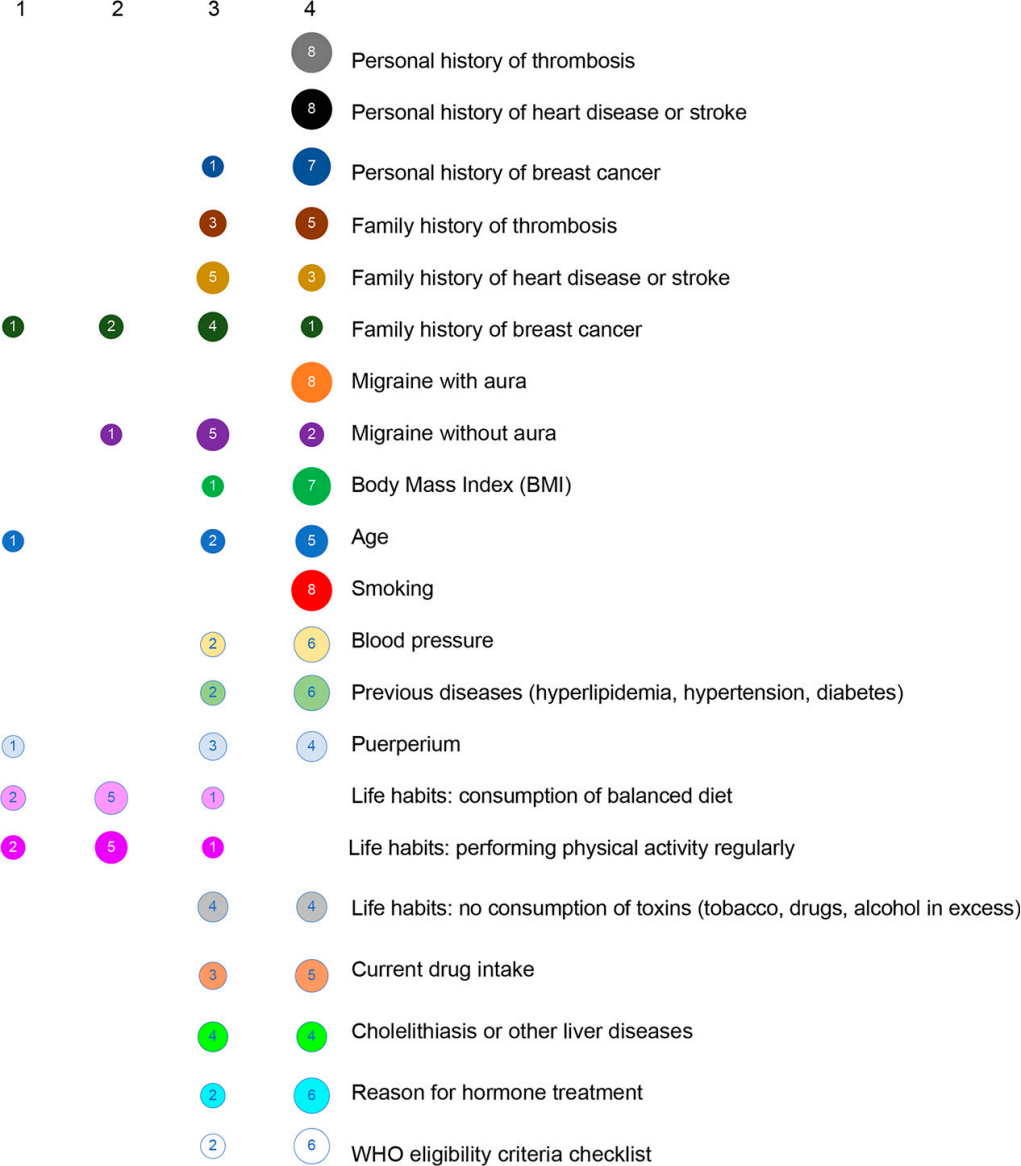
Importance of each variable during anamnesis. Four-point Likert scale scores: 1, unimportant; 2, somehow important; 3, important; 4, very important.

**FIG. 3.**
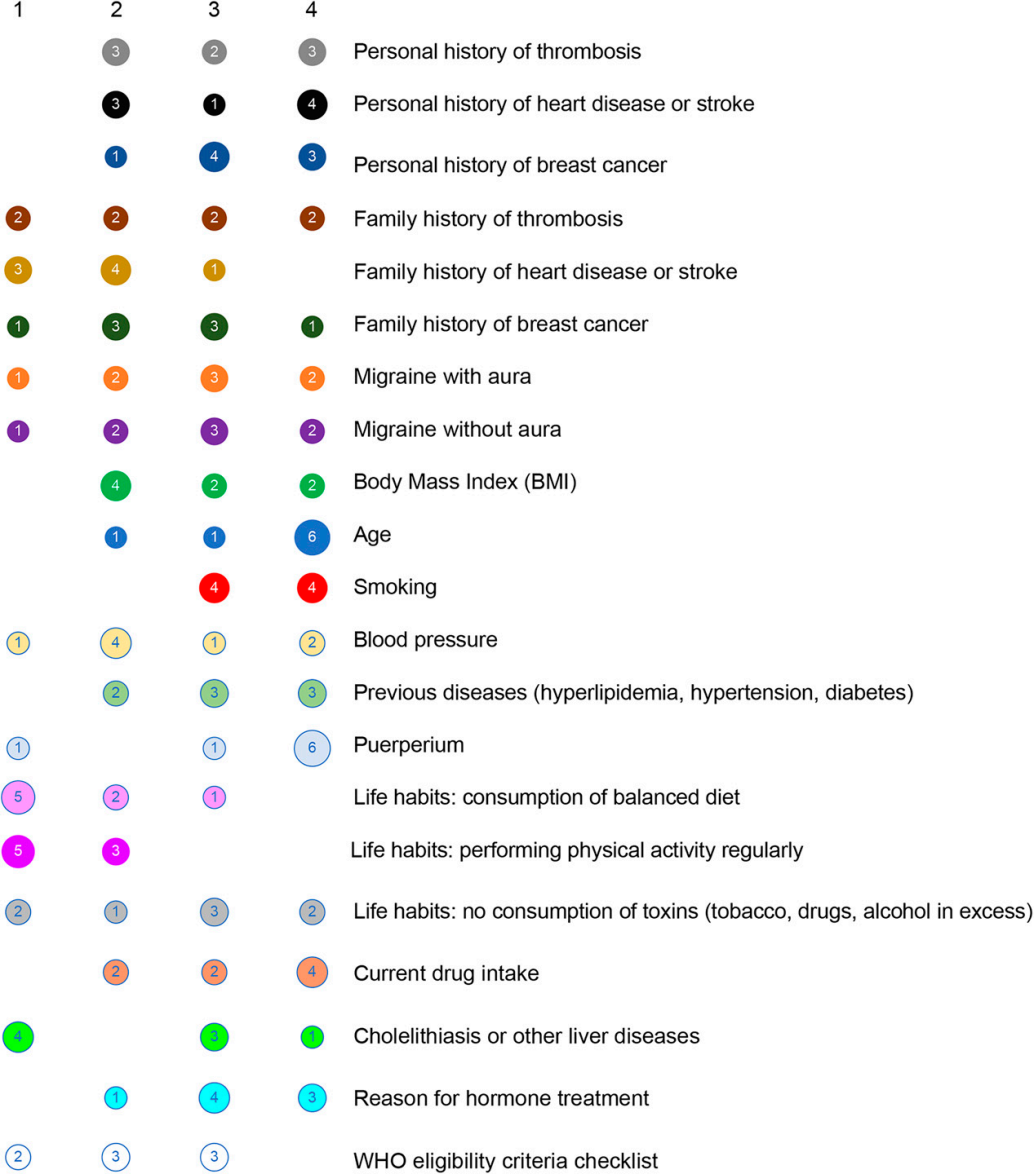
Frequency of each variable during anamnesis. Four-point Likert scale scores: 1, rare; 2: uncommon; 3, common; 4, very common.

The participants indicated that variables such as age, smoking, migraine with aura, personal history of thrombosis, and personal history of heart disease or stroke are considered important or very important (Fig. 2). However, participants also indicated that regardless of their importance, the variables may not be explored with the same frequency (Fig. 3). Moreover, puerperium was indicated as very frequently asked about (median score = 4), although the participants claimed that it is considered slightly less important (3.5).

**FIG. 4.**
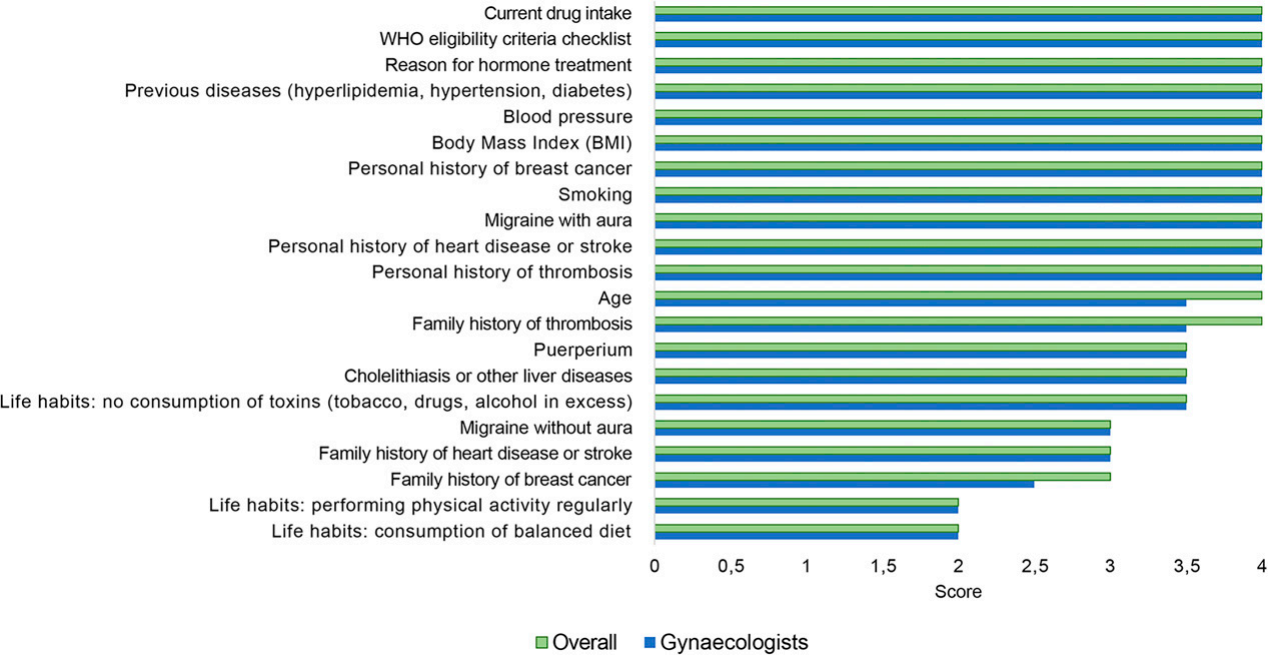
Overall median importance versus median importance according to gynecologists. Four-point Likert scale scores: 1, unimportant; 2, somehow important; 3, important; 4, very important.

**FIG. 5.**
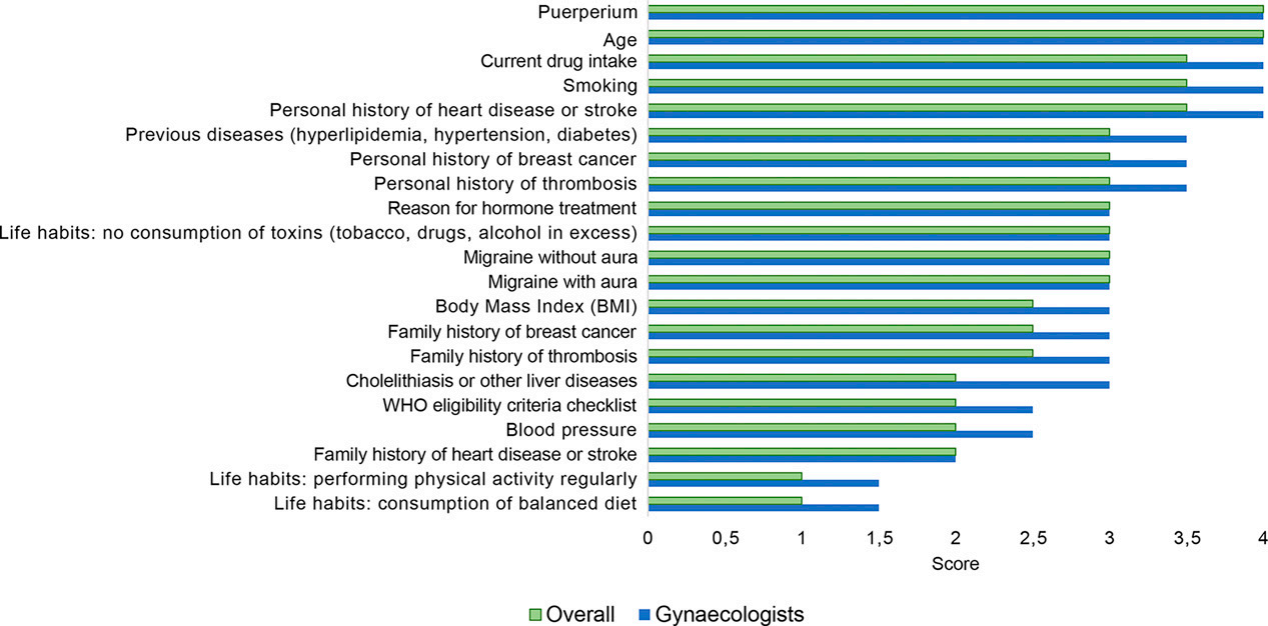
Overall median frequency versus median frequency according to gynecologists. Four-point Likert scale scores: 1, rare; 2: uncommon; 3, common; 4, very common.

Numerical differences were observed between internists and gynecologists, with more importance given to the following items by internists: age (median score = 3.5), family history of breast cancer (2.5), and family history of thrombosis (3.5), as presented in Figure 4. On the contrary, gynecologists gave numerically higher scores to the frequency of most variables (Fig. 5), but according to the internists, some variables are asked about less often than what the gynecologists claimed, such as BMI, personal history of thrombosis, and smoking.

Based on the median scores obtained for the importance of each item, the participants’ experience, and the available literature, a checklist with 19 items was defined. The checklist, which is presented in Table 1, includes the items classified into five groups: general items regarding the characteristics of the user, personal history and family history of diseases that are considered risk factors, migraine with or without aura, and other diseases that would be relevant for the OC recommendation. Moreover, we included two columns named “Checked” and “Comments” to provide a general overview to the gynecologist performing the anamnesis.

**Table 1.** Anamnesis Checklist for Oral Contraceptive Choice

Variable	Checked	Comments
General items		
Reason for treatment		
Age		
BMI		
Smoking status		
Blood pressure		
Puerperium/lactation		
Personal history		
Thrombosis		
Heart disease or stroke		
Breast cancer		
Family history		
Thrombosis		
Heart disease or stroke		
Breast cancer		
Migraine		
With aura		
Without aura		
Other diseases		
Hyperlipidemia		
Diabetes		
Cholelithiasis		
Other liver diseases		
Drug or alcohol intake		

BMI, body mass index.

## Discussion

The prescription of contraceptive drugs should be based on safety for users, aiming the lowest possible risk. This anamnesis guide seeks to help health care professionals when it comes to OC prescriptions.

Potential users should be informed about the balance between risks and benefits of the different options offered, and recommendations should be adapted to the user’s needs along their lives as their needs change over time. The physicians also highlighted that, contrary to popular belief, POP and COC provide similar protection, thus efficacy should not be a factor altering the choice of OC. However, the safety profile differs and should be the main characteristic considered, as COC can be associated with a higher risk of VTE and other adverse events.^1–5^ Furthermore, since one of the user’s concerns with POP is the cycle control and the presence of unscheduled bleeding, it is important to emphasize to them that these bleedings are not associated with decreased effectiveness and are usually resolved over time.^8,15,16,27^ Nevertheless, depending on the reason for starting OC, the estrogens present in COC may be required to achieve the expected results. In these situations, natural estrogens may be considered as they are associated with a lower risk of VTE than synthetic estrogens.^28^

The physicians claimed that age is viewed as less relevant during anamnesis, as it is a transversal factor always considered independent from the reason for consultation. Moreover, although the prevalence of VTE increases with age,^29^ during the discussion, the gynecologists participating in the study described several cases in young users, highlighting that other factors may be more important than age. Nevertheless, POP could be recommended as first choice for individuals above 35 years of age, if risk factors are present. However, if estrogens are required, the choice should be the estrogen preparation with the lowest impact on cardiovascular risk.^30^ The participants highlighted the recommendation of COC for particular situations such as ovarian failure, polycystic ovary syndrome, amenorrhea, hirsutism, or von Willebrand disease. Therefore, all agreed that POP should be recommended for those above 50 years and for those who would not require estrogens. This highlights the importance of the user’s needs and the reason to start an OC as factors that should be evaluated during counseling.

Two key variables are BMI and smoking status. Both were considered very important and frequently taken into account, in line with previous studies reporting an 11.63-fold increased risk of VTE in overweight users and a 23.78-fold increase in obese users versus those with a normal BMI.^31^ Several studies regarding smokers’ status report an 8.8-fold increased risk for smokers using OC compared with never-smokers not using OC.^32^ Surprisingly, blood pressure was rarely evaluated, although considered very important. The participants highlighted that blood pressure should always be evaluated before choosing an OC, as COC are contraindicated for users with high blood pressure.^24,33^

It is also notable that internists gave lower scores to the frequency of evaluation of migraine (with or without aura) and puerperium, although these scores did not alter the overall median. However, all participants agreed during the work sessions that these factors are very important and should be evaluated. Overall, although migraines with aura were considered very important by the participants, they claimed that this variable is not always asked about. Migraines with aura have been associated with an increased risk of ischemic and hemorrhagic stroke (RR = 1.55), especially for smokers, and therefore COC are contraindicated in users with migraine with aura.^34,35^ Furthermore, puerperium is a period in which the baseline risk of VTE is increased (RR = 4.29) and the management of estrogen-containing hormonal contraceptives should be managed with care.^36,37^ Moreover, the participants highlighted that lactation should be considered together with puerperium to decide, which would be the best option and the ideal timing.^38^

The gynecologists indicated that, in their opinion, the family history of thrombosis or breast cancer is considered less important, as the personal history would be more relevant.^39^ Similarly, other conditions were considered very important but not often evaluated. Given that contraceptives can impact the lipid profile^40^ and diabetes is considered a risk factor for VTE,^41^ these conditions should be evaluated before recommending OC. Moreover, the immunomodulatory effect of OCs in the liver should be taken into account,^42^ and it is important to consider it before counseling, although it is not always present during anamnesis.

As described above, the reasons for hormone treatment play an important role and should be part of the anamnesis to decide if the estrogens present in COC are needed as part of the treatment. Moreover, individual risk factors should always be evaluated and periodically revised, while increasing the awareness about signs and symptoms of VTE. Therefore, POP should be presented as an option even if there is no clear risk factor, as the risk of VTE is always present. The usage trend of COCs has decreased within the European setting, shifting toward POP, whose aim is to use safer contraceptive forms.^43,44^ The recommendation of POP should be accompanied by an explanation of their benefits, and being clear about the expectations could increase the acceptability and satisfaction with these prescriptions, as not knowing the benefits is a factor to avoid these treatments. Being aware of these particularities could increase treatment persistence, but considering that the risk factors of VTE and the expectations can change over time,^45^ a regular follow-up was considered indispensable, as the situation should be periodically re-evaluated and, if required, switch to another OC.

### Strengths and limitations

The design-thinking methodology is one of the main strengths of the study, involving participants from different regions and settings. The checklist produced presents a comprehensive approach, developed by experts from different areas and based on current evidence. In addition, it is structured into five clear categories that ease its implementation in routine clinical practice. Moreover, it is a flexible checklist, adaptable to individual needs but unifying the approach to anamnesis. However, one weakness is the limited number of participants, the fact that all are located in Spain, and the lack of family physicians and midwifes among the participants. Moreover, the participants individually scored the variables based on their perceptions, but their answers could be biased by their own clinical practice. Similarly, the user perception is limited to the impressions of the participants and could be directly collected in future studies. It is also worth noting the lack of external validation in different clinical contexts, as well as the possible lack of variables that could be relevant and have not been taken into account by the participants. Future studies will be required to test and validate this proposed anamnesis checklist.

## Conclusions

The study highlights the most relevant variables that should be analyzed for a good anamnesis prior to counseling and prescription. For this purpose, a checklist has been developed to help the clinician to safely prescribe an OC.

## Abbreviations Used

BMI: body mass index
COC: combined oral contraceptive
DRSP: drospirenone
OC: oral contraceptive
POP: progestin-only pills
VTE: venous thromboembolism
