# Supplementary material for: Anamnesis Checklist for Informed Oral Contraceptive Choices: A Spanish Perspective
Source: Womens Health Rep (New Rochelle). 2025 Jan 13;6(1):50–9. doi: 10.1089/whr.2024.0073 (PMC11773164; doi:10.1089/whr.2024.0073)
Supplement: Supplementary Table S1 [file whr.2024.0073_supp_tables1.docx]

Supplementary Table 1: Survey variables

| **Variables** | **Priority**  **(1-4)** | **Frequency**  **(1-4)** |
| --- | --- | --- |
| Personal history of thrombosis |  |  |
| Personal history of heart disease or stroke |  |  |
| Personal history of breast cancer |  |  |
| Family history of thrombosis |  |  |
| Family history of heart disease or stroke |  |  |
| Family history of breast cancer |  |  |
| Migraine with aura |  |  |
| Migraine without aura |  |  |
| Body Mass Index (BMI) |  |  |
| Age |  |  |
| Smoking |  |  |
| Blood pressure |  |  |
| Previous diseases (hyperlipidaemia, hypertension, diabetes) |  |  |
| Puerperium |  |  |
| Life habits: consumption of balanced diet |  |  |
| Life habits: performing physical activity regularly |  |  |
| Life habits: no consumption of toxins (tobacco, drugs, alcohol in excess) |  |  |
| Current drug intake |  |  |
| Cholelithiasis or other liver diseases |  |  |
| Reason for hormone treatment |  |  |
| WHO eligibility criteria checklist |  |  |
